# Re-irradiation spine stereotactic body radiotherapy following high-dose conventional radiotherapy for metastatic epidural spinal cord compression: a retrospective study

**DOI:** 10.1007/s11604-024-01539-x

**Published:** 2024-02-28

**Authors:** Yutaro Koide, Shoichi Haimoto, Hidetoshi Shimizu, Takahiro Aoyama, Tomoki Kitagawa, Yurika Shindo, Naoya Nagai, Shingo Hashimoto, Hiroyuki Tachibana, Takeshi Kodaira

**Affiliations:** 1 Department of Radiation Oncology, Aichi Cancer Center Hospital, Kanokoden 1–1, Chikusa-Ku, Nagoya, Aichi Japan; 2 Department of Neurosurgery, Aichi Cancer Center Hospital, Chikusa-Ku, Nagoya, Japan

**Keywords:** Re-irradiation, Stereotactic radiotherapy, Spinal metastases, Spinal cord compression

## Abstract

**Purpose:**

We aimed to evaluate the efficacy and safety of re-irradiation stereotactic body radiation therapy (SBRT) in patients with metastatic epidural spinal cord compression (MESCC) following high-dose conventional radiotherapy.

**Materials and methods:**

Twenty-one patients met the following eligibility criteria: with an irradiation history of 50 Gy_2_ equivalent dose in 2-Gy fractions (EQD2) or more, diagnosed MESCC in the cervical or thoracic spines, and treated with re-irradiation SBRT of 24 Gy in 2 fractions between April 2018 and March 2023. Prior treatment was radiotherapy alone, not including surgery. The primary endpoint was a 1-year local failure rate. Overall survival (OS) and treatment-related adverse events were assessed as the secondary endpoints. Since our cohort includes one treatment-related death (TRD) of esophageal perforation, the cumulative esophageal dose was evaluated to find the dose constraints related to severe toxicities.

**Results:**

The median age was 68, and 14 males were included. The primary tumor sites (esophagus/lung/head and neck/others) were 6/6/7/2, and the median initial radiotherapy dose was 60 Gy_2_ EQD2 (range: 50–105 Gy_2_, 60–70/ > 70 Gy_2_ were 11/4). Ten patients underwent surgery followed by SBRT and 11 SBRT alone. At the median follow-up time of 10.4 months, 17 patients died of systemic disease progression including one TRD. No radiation-induced myelopathy or nerve root injuries occurred. Local failure occurred in six patients, with a 1-year local failure rate of 29.3% and a 1-year OS of 55.0%. Other toxicities included five cases of vertebral compression fractures (23.8%) and one radiation pneumonitis. The cumulative esophageal dose was recommended as follows: D_max_ < 203, D_0.035 cc_ < 187, and D_1cc_ < 167 (Gy_3_ in biological effective dose).

**Conclusion:**

Re-irradiation spine SBRT may be effective for selected patients with cervical or thoracic MESCC, even with high-dose irradiation histories. The cumulative dose assessment across the original and re-irradiated esophagus was recommended to decrease the risk of severe esophageal toxicities.

**Supplementary Information:**

The online version contains supplementary material available at 10.1007/s11604-024-01539-x.

## Introduction

Metastatic epidural spinal cord compression (MESCC) is a major concern but a clinical challenge for the management of advanced cancer patients, leading to significant morbidity, including pain, loss of neurologic function, and poor quality of life. Surgery followed by radiotherapy (RT) or RT alone is recommended for patients presenting with spinal cord compression [1–4]. Especially patients with radiosensitive tumors (e.g., lymphoma, myeloma, small cell lung cancer, germ cell tumor, prostate cancer, and breast cancer) or not severe spinal instability is well expected for pain relief, neurological recovery, and local tumor control even with radiotherapy alone [1, 4, 5].

During follow-up after the initial treatment, salvage treatment is considered if symptomatic or radiographic progression or recurrence is detected [4]. Even if RT or surgery plus RT is performed as the initial treatment, salvage RT is effective [6, 7]. Stereotactic body radiotherapy (SBRT) of the spine has recently emerged as an advanced RT technique expected to be highly effective in pain relief and tumor control [8–10]. Compared to conventional external beam radiotherapy (EBRT), SBRT has demonstrated effective local tumor control and safety due to its dose concentration and risk organ dose sparing [11–13]. International Stereotactic Radiosurgery Society practice guidelines suggest that SBRT can be a recommended treatment option for re-irradiation [14]. In cases of re-irradiation, it is vital to carefully account for the cumulative dose from current and previous treatments for proper risk evaluation and management [15, 16]. Previous reports on re-irradiation SBRT have only provided cases where the initial dose of EBRT was around 30 Gy in 10 fractions—few data exist about re-irradiation spine SBRT with a history of initial EBRT over 50 Gy equivalent dose in 2 Gy fractions (EQD2) [14]. In clinical practice, however, it is not uncommon for patients with a history of high-dose EBRT to undergo re-irradiation SBRT. Administering SBRT to patients with a history of high-dose EBRT carries a potential risk of severe adverse events related to radiation overdose, particularly in the cervical and thoracic areas, affecting critical organs like the esophagus, pharynx, and carotid artery [17–21]. Strict dose constraints and patient selection criteria are needed to minimize the risk of potentially fatal consequences.

We aimed to investigate the outcomes of re-irradiation SBRT for patients with progressive or recurrent spinal cord compression in the cervical and thoracic spines previously irradiated with 50 Gy_2_ EQD2 or more. The results of this study provide valuable data on the efficacy and safety of re-irradiation SBRT in such challenging situations and may contribute to the expansion of SBRT indications.

## Materials and methods

### Study design and data source

We retrospectively reviewed our institutional database and identified patients eligible for the following criteria: Patients with MESCC who previously received the conventional radiotherapy with a cumulative dose of 50 Gy2 EQD2 or more. Between April 2018 and March 2023, out of 136 patients treated with spinal SBRT, 21 met the following eligibility criteria: (1) cumulative prior EBRT dose ≥ 50 Gy to the target spines, and (2) re-irradiation of 24 Gy in two fractions of SBRT to the cervical and thoracic spines during the study period. Patients previously receiving SBRT at the current target spine as an initial treatment were excluded from the current study. The institutional ethical review board approved this study, and informed consent was obtained through an opt-out form option displayed on the website.

The patient follow-up for this study ended on September 30, 2023, and patients who were alive or lost follow-up were censored. The primary endpoint was defined as the local failure rate at 1 year. Local failure was defined according to the sc24 protocol as a gross unequivocal increase in tumor volume or linear dimension, any new or progressive tumor within the epidural space, and neurologic deterioration attributable to pre-existing epidural disease with equivocal increased epidural disease dimensions on CT/MRI [12]. As the secondary endpoints, we evaluated overall survival, defined as the survival time from the date of diagnosis of MESCC to the date of death or the last follow-up. Also, treatment-related adverse events in grade 2 or more were recorded and graded according to the Common Terminology Criteria for Adverse Events (CTCAE). Our cohort included one treatment-related death (TRD) of esophageal perforation; the esophageal dose was evaluated to find the biological effective dose (BED) limits related to severe toxicities.

### Patient selection criteria for re-irradiation SBRT

**Fig. 1:**
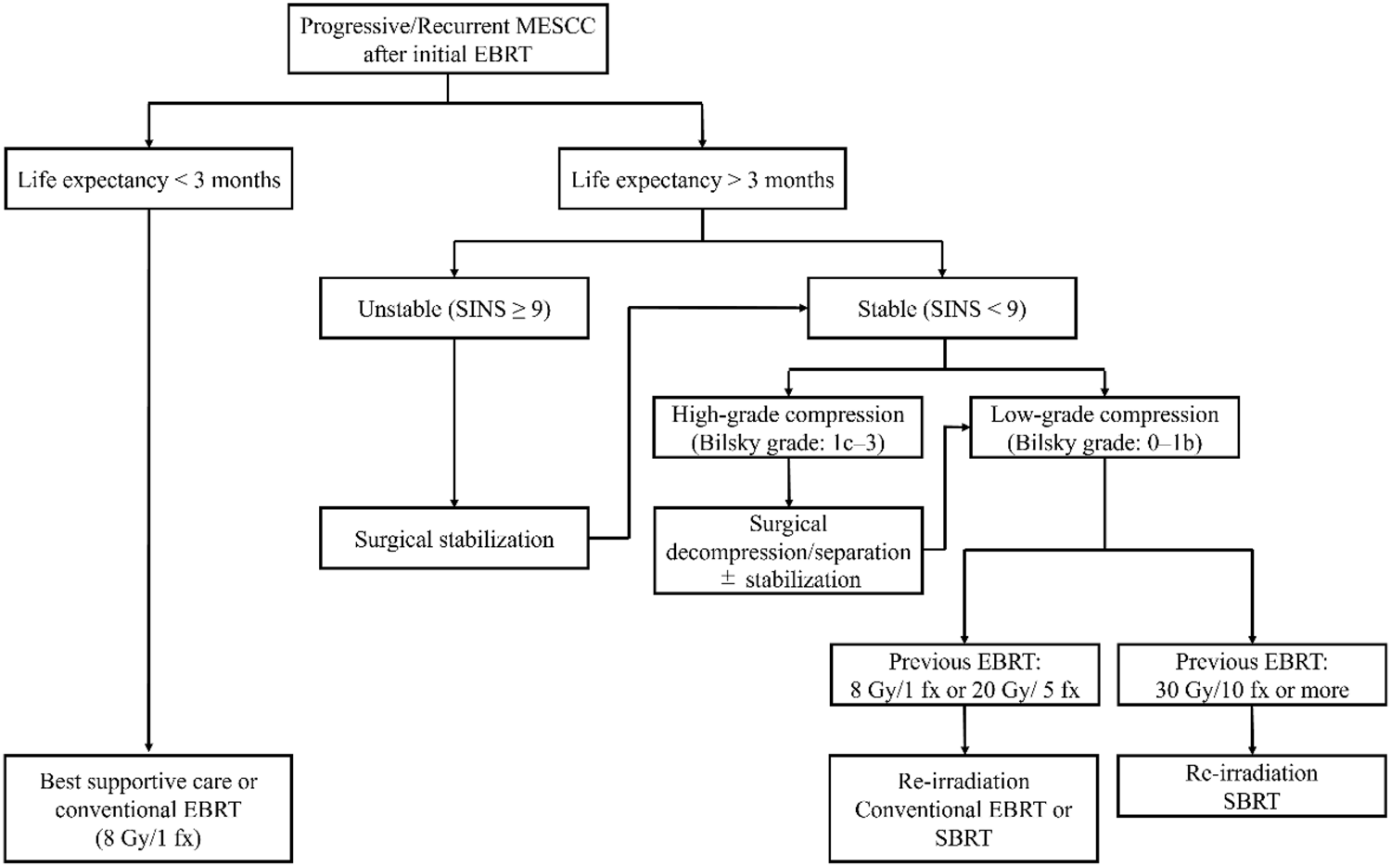
Algorithm for management of progressive or recurrent spinal cord compression received by the initial conventional radiotherapy. *MESCC* metastatic epidural spinal cord compression, *EBRT* external beam radiation therapy, *SBRT* stereotactic body radiation therapy, *fx* fractions, *SINS* spinal instability neoplastic score

Patients with a history of initial EBRT more than 6 months before and diagnosed with MESCC, expected to survive for at least the next three months, were eligible for re-irradiation SBRT. Figure 1 shows the institutional treatment algorithm for MESCC patients with a history of initial EBRT. This algorithm was created in reference to the International Spine Consortium report [5]. A multidisciplinary approach involving spinal surgeons, radiation oncologists, and physicians for primary cancer was used to determine whether re-irradiation SBRT or surgery followed by re-irradiation SBRT was acceptable based on the severity of the spinal cord compression and spinal instability. All the patients involved in this study were assessed before treatment. The severity of the cord compression is determined by Bilsky grade [22]: if grade 0–1b (low grade), consider re-irradiation SBRT alone, and if grade 1c–3 (high grade), consider decompression/separation (± stabilization) followed by re-irradiation SBRT. The severity of the spinal instability was determined based on the Spine Instability Neoplastic Score (SINS) [23]: if SINS < 9 (low-grade instability), consider re-irradiation SBRT alone, and if SINS is 9 or more (high-grade instability), consider stabilization (± decompression) followed by re-irradiation SBRT. If surgery was considered but not medically possible, only re-irradiation SBRT was performed. After treatment, all patients were followed up every 2–3 months using CT or MRI.

### Treatment planning for SBRT

Patients were immobilized in a supine position using an immobilization device (a vacuum bag for the thoracic spine and a thermoplastic head-neck mask for the cervical to upper thoracic spine). Treatment planning CT was performed with an Aquilion LB CT system (Canon Medical Systems, Tochigi, Japan), and CT slice thicknesses were 1 mm (pixel size 512 × 512). MRI scans for fusion to the planning CT were obtained with patients immobilized in the same simulation position using the same device. A 1 mm slice of T1-, T2-weighted, and T1 post-gadolinium axial MRI images were obtained. The MRI images were used for delineating targets and the spinal cord in planning CT images. Postoperative patients with metal prostheses underwent CT myelogram: an intrathecal injection of iohexol contrast (Omnipaque 240; GE Healthcare, Princeton, New Jersey, USA) was performed by the neurosurgeon 2 h before planning CT simulation. To spread the contrast medium around the target spine, injected patients are placed in a high pelvic position until CT scanning.

The gross tumor volume (GTV) was defined as the disease visible on CT and fused MRI images. The clinical target volume (CTV) was delineated based on the international consortium guidelines [24, 25]. The guidelines recommend using the proposed anatomic classification system, which divides each vertebral body into six sectors (the vertebral body, the left/right pedicles, the left/right transverse processes and laminas, and the spinous process). CTV contour generation was determined depending on which sectors GTV was involved in. The entire sector was included in the CTV if any portion of these regions contained the GTV. Additionally, the sectors next to the GTV-involved sectors on both sides were included in the CTV (e.g., If the vertebral body is involved with GTV the entire vertebral body, and the left and right pedicles are included in the CTV). A uniform margin of 2 mm with CTV is required for planning target volume (PTV). We defined PTV_eval_ as the volume obtained by subtracting the spinal cord PRV from the PTV. The prescription was set at 24 Gy in two fractions (12 Gy per fraction) for PTV_eval_, delivered daily on Monday–Friday. The beam delivery technique was used volumetric modulated arc therapy (VMAT) in a flattening filter-free mode using 10-MV photon on the Varian TrueBeam system (Varian Medical Systems, Palo Alto, CA). The treatment planning was RayStation version 10.0 (RaySearch Laboratories) using the collapsed cone dose algorithm for dose calculation. Table S1 shows dose specification for PTV_eval_.

Organs at risk (OAR) in this study included the spinal cord, pharynx, larynx, esophagus, trachea, main bronchus, stomach, lungs, carotid arteries, and aorta. The spinal cord was delineated based on T2-weighted MRI fused to the planning CT. In case of postoperative patients with metal prostheses, we used CT myelogram for spinal cord delineation [26, 27]. The other OARs were delineated based on the planning CT alone. Planning organ at risk volume (PRV) margin of 1.5 mm is required for the spinal cord.

Table S2 shows dose constraints for specified OARs. The maximum dose of 0.035 cc (D_0.035 cc_) to the spinal cord PRV was determined with the highest priority based on prior EBRT dose (BED ≤ 90 Gy_2_, 90–100 Gy_2_, > 100 Gy_2_): D_0.035 cc_ ≤ 12.2 Gy in case of prior EBRT dose ≤ 90 Gy_2_ in BED, D_0.035 cc_ ≤ 10.8 Gy in case of prior EBRT dose > 100 Gy_2_ in BED. For past EBRT doses of 90–100 Gy_2_ in BED, D_0.035 cc_ was calculated based on the report of Sahgal et al., assuming a linear fall from 12.2 Gy to 10.8 Gy (e.g., D_0.035 cc_ ≤ 11.4 Gy in the case of 95 Gy_2_ < BED ≤ 96 Gy_2_). D_0.035 cc_ of the other specified OARs and unspecified tissues should be ≤ 20 Gy.

### Post-SBRT dosimetric analysis and statistical analysis

This study strongly required the spinal cord and other OARs to adhere to dose constraints. The cumulative doses of the esophagus, spinal cord, and other specified OARs were evaluated retrospectively. The cumulative doses of the SBRT and the initial EBRT plans were calculated using MIM Maestro (MIM Software Inc., Cleveland, USA): D_max_, D_0.035 cc_, and D_1cc_ of each specified OAR with α/β = 2, 3, and 10 were displayed in the EQD2 and BED dose, respectively. The cumulative doses may be low if some organs are distant from the target vertebrae or surgically removed or implanted, and not all OAR doses are evaluated in each patient. The criteria for inclusion in the dosimetric analysis were a maximum cumulative dose of 50 Gy_2_ EQD2 or more for the spinal cord (12 patients), 60 Gy_2_ EQD2 or more for the esophagus, trachea, and aorta (15, 15, and 17 patients), and 66 Gy or more for the carotid artery and pharynx (12 and 7 patients).

Survival analysis was performed using the Kaplan–Meier method, and differences between survival curves were assessed using the log-rank test. Local failure was estimated using cumulative incidence functions, accounting for death without tumor recurrence as a competing risk. In additional concern, local failure and survival were compared between patients distinguished with the oligo-recurrence disease or not using the following criteria: (1) one to several distant metastases/recurrences in one to several organs, (2) primary site of the cancer controlled, (3) one to several distant metastases/recurrences can be treated with local therapy, and (4) no other distant metastases/recurrences other than those in (3) [28–30]. A p value less than 0.05 was considered statistically significant. All statistical analyses were performed using R statistical software version 4.2.2 (The R Foundation for Statistical Computing, Vienna, Austria).

## Results

### Patient

Table 1 shows the patient characteristics. The 21 eligible patients included 14 males and 7 females with a median age of 68 (interquartile range, IQR: 56–72). Each patient had an initial EBRT history of median 60 Gy/30 fractions (120 Gy_2_ BED, range: 100–210 Gy_2_ BED), including 15 patients (71%) who received more than 60 Gy/30 fractions (120 Gy_2_ BED) and 4 patients (19%) with more than 70 Gy/35 fractions (140 Gy_2_ BED). Fifteen patients received initial EBRT as definitive and six patients as palliative therapy. Five patients had more than two courses of previous irradiation treatments and were categorized based on their cumulative doses. At the time of diagnosis of recurrent MESCC, 17 patients (81%) had neurologic symptoms or pains, and 4 (19%) were asymptomatic but diagnosed based on radiological findings. The median interval between the last EBRT and recurrent MESCC diagnosis was 13.7 months (IQR: 8.6–30.8 months).

**Table 1:** Patient characteristics

Factor	Overall (*N* = 21)
Age	
Median [IQR]	68 [56, 72]
Sex (%)	
Female	7 (33.3)
Male	14 (66.7)
ECOG performance status (%)	
0	4 (19.0)
1	15 (71.4)
2	0
3	2 (9.5)
4	0
Primary lesion (%)	
Esophageal cancer	6 (28.6)
Head and neck cancer	7 (33.3)
Lung cancer	6 (28.6)
Others (Hepatocellular carcinoma, Sarcoma)	2 (9.5)
Initial RT prescription dose	
50–60 Gy_2_ EQD2	6 (28.6)
60–70 Gy_2_ EQD2	11 (52.4)
≥ 70 Gy_2_ EQD2	4 (19.0)
Global maximum dose	
Maximum dose [IQR]	90.6 [63.0, 72.3], Gy_2_ EQD2
Spinal cord maximum dose	
Maximum dose [IQR]	53.4 [31.8, 47.9], Gy_2_ EQD2
< 40 Gy_2_ EQD2	12 (57.1)
40–45 Gy_2_ EQD2	1 (4.8)
45–50 Gy_2_ EQD2	5 (23.8)
≥ 50 Gy_2_ EQD2	3 (14.3)
Esophageal maximum dose*	
Maximum dose [IQR]	72.6 [51.7, 63.1], Gy_2_ EQD2
< 50 Gy_2_ EQD2	3 (18.8)
50–60 Gy_2_ EQD2	4 (25.0)
60–70 Gy_2_ EQD2	8 (50.0)
≥ 70 Gy_2_ EQD2	1 (6.2)
Symptom at diagnosis of recurrent MESCC	
Symptomatic	17 (81.0)
Asymptomatic (Diagnosis by radiographic imaging)	4 (19.0)
Surgery	
Surgery followed by SBRT	10 (47.6)
SBRT alone	11 (52.3)
Interval between initial RT and SBRT, months	
Median [IQR]	13.7 [8.6, 30.8]
Target spinal levels (%)	
Cervical spine (C1–5)	3 (14.3)
Cervical-thoracic junctional spine (C6–Th3)	8 (38.1)
Thoracic spine (Th4–Th12)	10 (47.6)
Number of target vertebrae (%)	
1	10 (47.6)
2–3	8 (38.1)
≧ 4	3 (14.3)
Systemic disease	
Controlled	6 (28.6)
Active	15 (71.4)
SINS (%)	
0–6 (stable)	7 (33.3)
7–12 (potentially unstable)	7 (33.3)
13–18 (unstable)	7 (33.3)
Bilsky grade (%)	
0 (Bone only disease)	3 (14.3)
1a (Epidural impingement, without thecal sac deformation)	4 (19.0)
1b (Thecal sac deformation, without spinal cord abutment)	5 (23.8)
1c (Spinal cord abutment, without compression)	3 (14.3)
2 (Spinal cord compression, CSF visible)	3 (14.3)
3 (Spinal cord compression, no CSF visible)	3 (14.3)
Frankel classification (%)	
E (normal motor)	18 (85.7)
D (preserved motor function)	1 (4.8)
C (preserved motor non-functional)	2 (9.6)

*IQR* interquartile range, *ECOG* eastern cooperative oncology group, *EQD2* equivalent dose at 2 Gy, *MESCC* metastatic epidural spinal cord compression, *SBRT* stereotactic body radiation therapy, *SINS* spinal instability neoplastic score, *CSF* cerebrospinal fluid

*The esophageal doses at initial RT for 16 patients are shown, excluding five patients who had primary head and neck cancer or underwent esophagectomy

Eleven patients (52.4%) received SBRT alone, and 10 (47.6%) received SBRT following surgery: six received decompression plus stabilization surgery, and four received stabilization surgery. Although four patients in the SBRT alone group had met the institutional criteria for surgery depending on their spinal instability and spinal cord compression, they were considered non-surgical candidates due to poor performance status (*N* = 1) or risk of discontinuing systemic therapy for advanced systemic disease (*N* = 3). All SBRT plans achieved the pre-specified institutional dose constraints (Table S1–2), and all patients completed SBRT on schedule.

### Local failure rate and survival

**Fig. 2:**
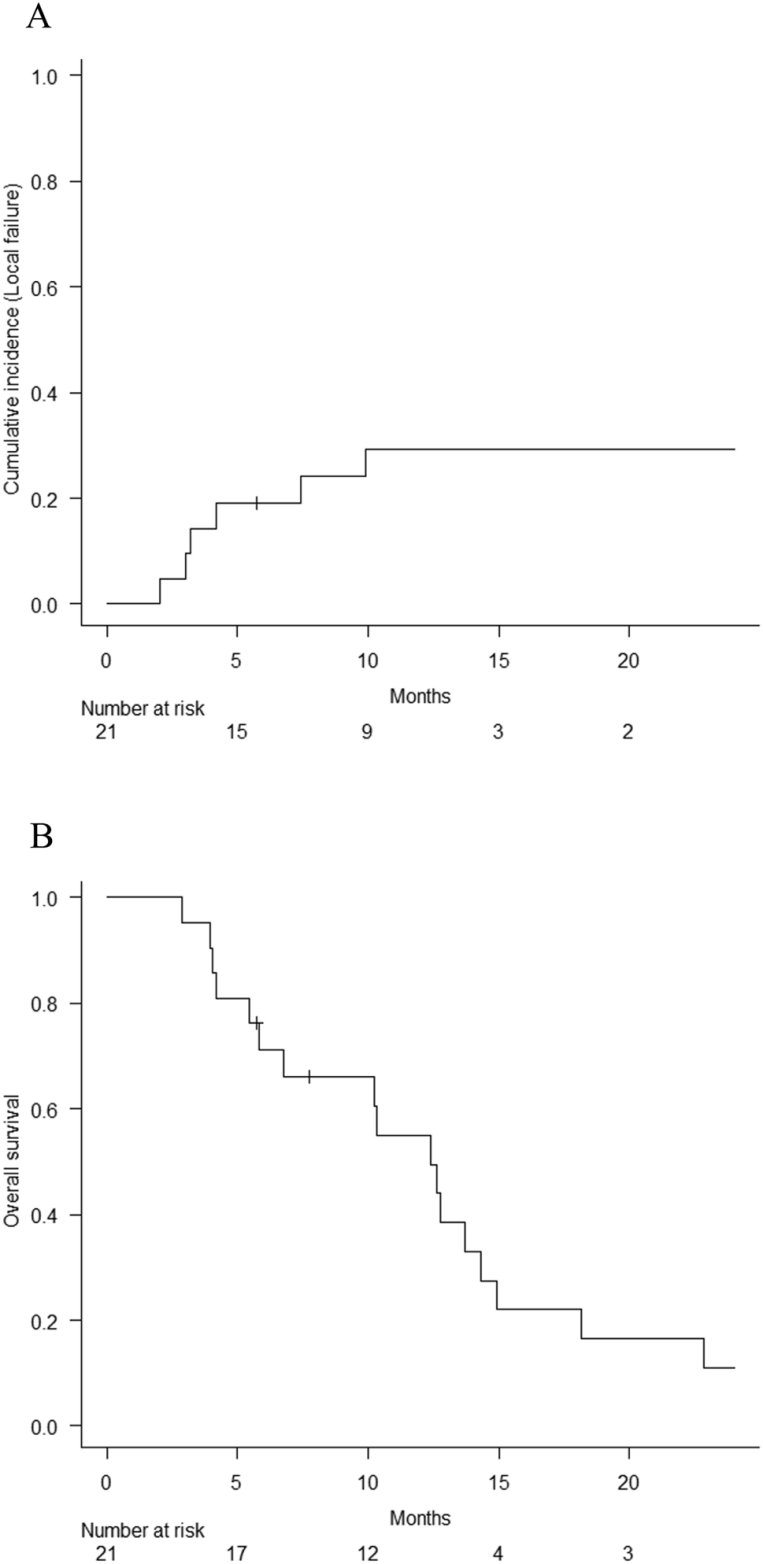
Local failure rate and overall survival in the entire cohort

The median follow-up time from the diagnosis of recurrent or progressive spinal metastases was 10.4 months (range: 2.9–57.5 months). Seventeen patients died at a median of 10.3 months (2.9–22.8), and the median follow-up time of 4 alive patients was 18.0 months (5.8–57.5 months). As shown in Fig. 2A, the median survival time was 12.4 months (95% CI 5.8–14.3), and the 1-year overall survival rate was 55.0% (95% CI 31.1–73.7). Until the last follow-up, the local tumor was controlled in 15 patients (71%), and six experienced local failures: 1 in the surgery followed by the SBRT group and 5 in the SBRT alone group. The 1-year local failure rate was 29.3% (95% CI 11.4–50.0) in the entire cohort (Fig. 2B). All four unstable but non-surgical patients developed early local failure (median 3.7 months to failure, range 2.0–7.4). If the cohort excluded them, 1-year local failure rate was 12.3% (95% CI 1.8–33.3). As for the oligo-recurrence subgroup, one patient (20%) out of 5 oligo-recurrence patients experienced local recurrence; in contrast, 5 local recurrences (31%) in the other 16 patients. Although it was not statistically significant, the oligo-recurrence patients tended to have longer survival (MST: 10.3 months vs. 18.2 months, *P* = 0.084).

Of the 6 patients with local failure, 5 received additional salvage treatment: surgery in 2 patients, surgery plus repeat SBRT in 1, and repeat SBRT alone in 2. Although 3 patients achieved local tumor control with avoided neurological deficiency, 2 developed progressive disease with leptomeningeal dissemination. One patient was in poor general condition and was administered supportive care without salvage treatment.

### Adverse events

**Fig. 3:**
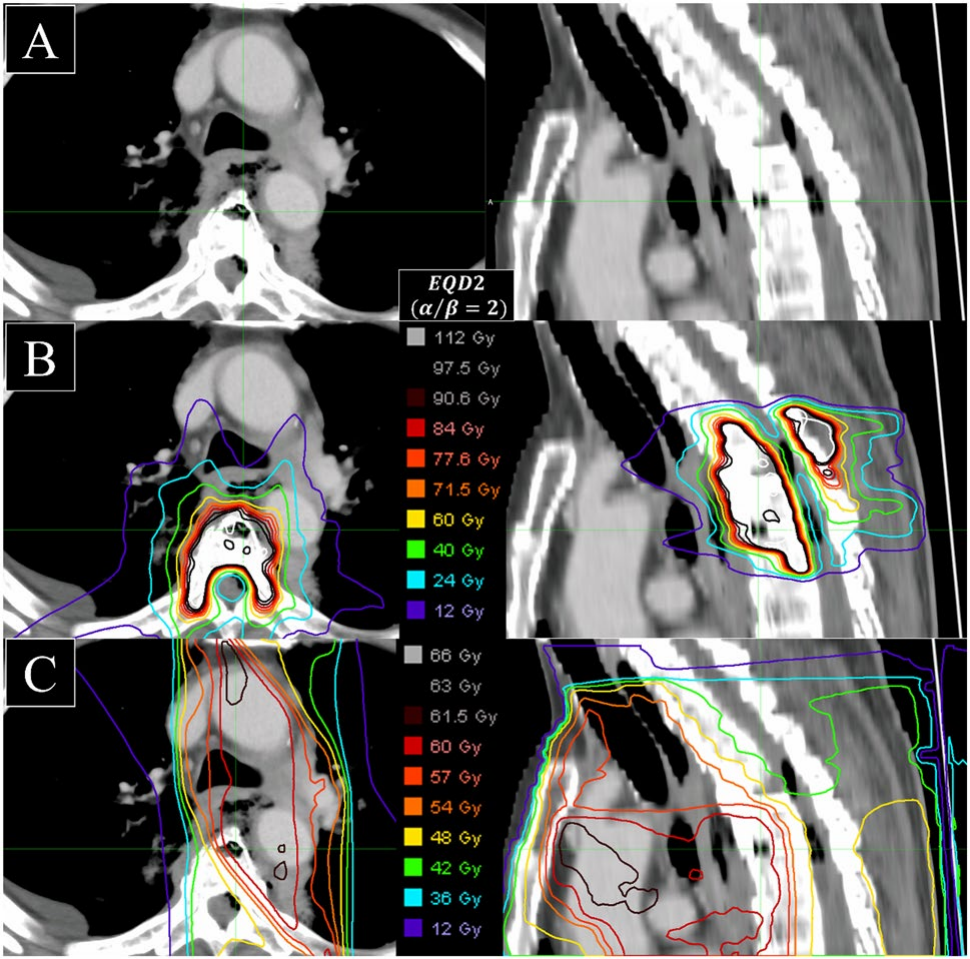
The images of diagnostic CT of radiation-induced esophageal perforation. **A** The diagnostic image. The patient was urgently hospitalized 18 days before death due to a fever and developed paraplegia 12 days before death. The image shows esophageal perforation, mediastinitis, and spinal cord compression due to an epidural abscess. **B** Dose distribution of re-irradiation SBRT on the fused-CT image (same CT as Fig. 2A). **C** Dose distribution of initial EBRT on the fused-CT image (same CT as Fig. 2A). *CT* computed tomography, *SBRT* stereotactic body radiation therapy, *EBRT* external beam radiation therapy

Table 2 summarizes grade 2–5 radiation-induced adverse events. Treatment-related mortality occurred in 1 patient (4.8%) due to mediastinitis and epidural abscess from esophageal perforation. The patient who died had active systemic disease due to recurrent esophageal cancer and received re-irradiation SBRT for Th4–5 MESCC (Bilsky grade 1b and SINS 5) 7.8 months after the initial chemoradiotherapy (60 Gy in 30 fractions). Two weeks after SBRT, systemic chemotherapy (5-fluorouracil and cisplatin) was resumed. After the systemic disease progression, the treatment was switched to weekly paclitaxel until 25 days before death. The patient was urgently hospitalized 18 days before death due to a fever and developed paraplegia 12 days before death. CT scans (Fig. 3A) diagnosed esophageal perforation, mediastinitis, and spinal cord compression due to an epidural abscess (Esophageal perforation diagnosed 154 days after SBRT). Initially, the esophageal perforation was attributed to the progression of the mediastinal tumor. However, fusing the dose distributions of initial EBRT and SBRT to the diagnostic CT (Fig. 3A) showed that the esophageal perforation occurred where the location irradiated high doses in both plans (Fig. 3B-C). Eventually, the death was highly suspected to be a treatment-related death (TRD) due to an overdose of esophagus from SBRT. The patient died 166 days after the diagnosis of the spinal metastases and 124 days after SBRT.

**Table 2:** Incidence of grade 2 or higher treatment-related adverse events

Adverse events	Overall (*N* = 21)				
	Grade 2–5	Grade 2	Grade 3	Grade 4	Grade 5
Dysphagia	2 (10%)	1 (5%)	1 (5%)	0	0
Nausea	2 (10%)	1 (5%)	1 (5%)	0	0
Esophagitis	2 (10%)	1 (5%)	0	0	1 (5%)
Vertebral compression fracture	5 (24%)	3 (14%)	2 (10%)	0	0
Radiation pneumonitis	1 (5%)	1 (5%)	0	0	0

Other identified adverse events included vertebral compression fractures (VCF) of any grade in 5 (23.8%) of the entire patients: they were all treated with SBRT alone, including 3 of 4 patients (75%) who had spinal instability but not performed surgery prior to SBRT. Two patients with grade 3 VCF underwent stabilization. Radiation pneumonitis (grade 2) occurred in one patient (4.8%). No cases of radiation-induced myelopathy or nerve root injury or carotid artery rupture were observed.

### Dosimetric analyses

**Fig. 4:**
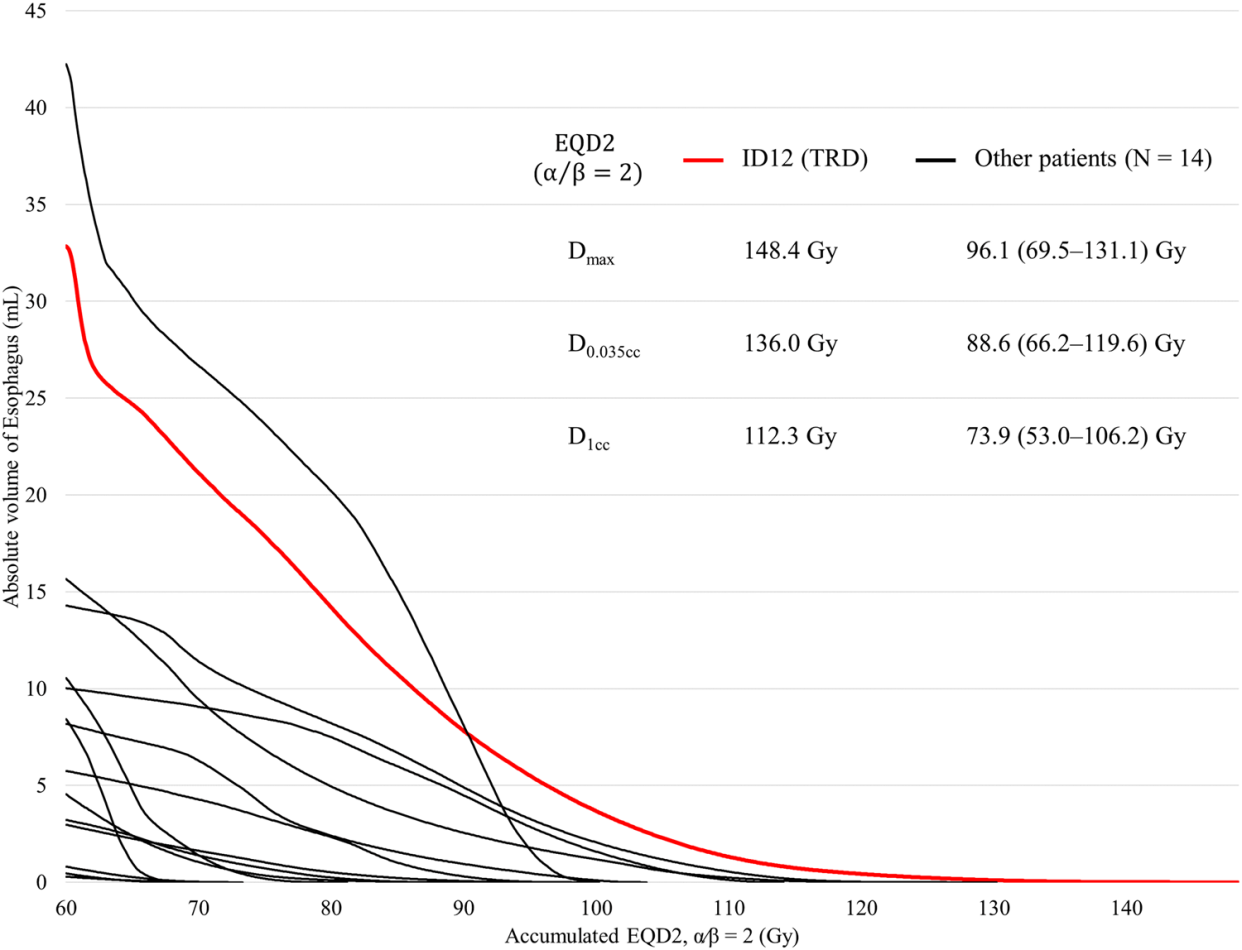
A Dose volume histogram for cumulative esophageal dose (equivalent dose at 2 Gy with the α/β ratio of 2). Red line shows a histogram of the patient died of radiation-induced esophageal perforation (grade 5), and Black lines show those of the other patients

The esophagus of the TRD patient was irradiated with a higher dose per volume for both SBRT alone and the cumulative dose with initial EBRT. Table 3 shows the Dmax, D0.035 cc, and D1cc, which were higher than the maximum values compared to the TRD patient (identification number 12) and other evaluable 15 patients: the SBRT dose was 127 Gy_3_ vs. 117 Gy_3_, 114 Gy_3_ vs. 106 Gy_3_, 83 Gy_3_ vs. 82 Gy_3_ in BED, and the cumulative dose was 227 Gy_3_ vs. 203 Gy_3_, 209 Gy_3_ vs. 187 Gy_3_, 175 Gy_3_ vs. 167 Gy_3_ in BED, respectively. Figure 4 shows the dose-volume histogram of the esophagus. In the TRD patient, the DVH shows that the cumulative esophageal dose curve extends into a higher dose range, showing a longer tail than in other patients.

**Table 3:** Esophageal dose comparison between the patient with grade 5 esophageal toxicity and other patients

	The TRD patient (*N* = 1) vs. other patients (*N* = 15)		
	D_max_	D_0.035 cc_	D_1cc_
SBRT dose, maximum value [IQR]			
EQD2			
α/β = 2, Gy2	89 vs. 82 [32, 66]	80 vs. 74 [25, 58]	57 vs. 57 [15, 48]
α/β = 3, Gy3	76 vs. 70 [28, 57]	68 vs. 64 [22, 50]	50 vs. 49 [14, 42]
α/β = 10, Gy10	46 vs. 43 [20, 36]	42 vs. 40 [16, 32]	32 vs. 32 [11, 28]
BED			
α/β = 2, Gy2	178 vs. 164 [63, 131]	160 vs. 148 [49, 116]	114 vs. 114 [29, 95]
α/β = 3, Gy3	127 vs. 117 [47, 95]	114 vs. 106 [37, 84]	83 vs. 82 [23, 69]
α/β = 10, Gy10	55 vs. 52 [24, 43]	51 vs. 48 [20, 39]	38 vs. 38 [13, 33]
Cumulative dose, maximum value [IQR]			
EQD2			
α/β = 2, Gy2	148 vs. 131 [76, 103]	136 vs. 120 [72, 101]	112 vs. 106 [66, 94]
α/β = 3, Gy3	136 vs. 122 [73, 96]	125 vs. 112 [70, 95]	105 vs. 100 [63, 90]
α/β = 10, Gy10	106 vs. 102 [74, 88]	100 vs. 97 [71, 87]	89 vs. 88 [65, 84]
BED			
α/β = 2, Gy2	296 vs. 262 [152, 206]	272 vs. 240 [144, 202]	224 vs. 212 [132, 188]
α/β = 3, Gy3	227 vs. 203 [121, 160]	209 vs. 187 [117, 158]	175 vs. 167 [104, 150]
α/β = 10, Gy10	127 vs. 122 [89, 106]	120 vs. 117 [85, 104]	107 vs. 106 [78, 101]

*EQD2* equivalent dose at 2 Gy, *BED* biological effective dose, *IQR* interquartile range, *SBRT* stereotactic body radiation therapy, *Cumulative dose* total dose accumulated SBRT dose and the initial external beam radiation therapy doses, *D*_*max*_ the maximum dose at one point of the target volume, *D*_*0.035 cc*_ the maximum dose that covered 0.035 cc of the target volume, *D*_*1cc*_ the maximum dose that covered 1 cc of the target volume

Twelve patients met the inclusion criteria for the post-SBRT dosimetric analysis of the esophagus: their prior irradiation included 4 patients of more than two irradiation courses and 8 patients of initial radiotherapy with 120 Gy2 BED prescription doses. Table S3 shows the cumulative doses of the spinal cord and other organs: the maximum value (IQR) of the spinal cord cumulative D_0.035 cc_ was 122 (104, 112) Gy_2_ BED. For other organs, pharynx, trachea, carotid arteries, and aorta, the maximum value (IQR) of the cumulative D_max_ was 200 (127, 173) Gy_3_ BED, 197 (111, 157) Gy_3_ BED, 197 (160, 178) Gy_3_ BED, and 373 (198, 271) Gy_3_ BED, respectively.

## Discussion

The results of our study demonstrate that re-irradiation with 24 Gy in 2 fractions of SBRT can be an effective salvage treatment for selected patients with cervical or thoracic MESCC previously irradiated with 50 Gy_2_ EQD2 or more. The analysis was limited to patients with a history of high-dose initial EBRT with a median 60 Gy_2_ EQD2 (α/β = 2, range: 50–105 Gy_2_), higher than those of any previous studies. Patients who receive high-dose initial EBRT may be at higher risk for overdose and severe toxicity of surrounding organs after salvage irradiation, because the surrounding organs are at least as or more irradiated than the spinal cord. The study has a strength in providing data on the efficacy and safety of salvage SBRT re-irradiation in cases of such challenging patients with a history of high-dose irradiation, such as the profile of radiation-induced toxicity and recommended esophageal dose constraints.

Our treatment algorithm shows the importance of patient selection for surgery plus SBRT or SBRT alone. A 1-year local failure rate of 29.3% highlights the possibility that SBRT can offer effective local control, even in patients with a history of high-dose radiation therapy. The current cohort had a higher incidence of treatment-related adverse events than in the previous studies, with 5 cases (24%) of VCF of any grade. As three (75%) non-surgical patients with unstable spinal lesions had VCF, the higher incidence of VCF was suspected to be due to the higher proportion of patients with high baseline spinal instability. Table S4 summarizes the previous studies of re-irradiation spine SBRT [31–43]. In the previous studies, the median initial EBRT dose was 30 Gy, and data on the efficacy and safety of cases with higher EBRT doses still need to be investigated. Ito et al. evaluated the efficacy and safety of re-irradiation SBRT (24 Gy/2 fx) stratified by previous EBRT doses (EQD2: < 30 Gy_2_, 30–40 Gy_2_, 40–50 Gy_2_, > 50 Gy_2_) [43]. The 1-year local failure rate was 25.8% in the entire cohort (123 patients), and the previous EBRT dose was not correlated with the local failure rate (*P* = 0.13). Their cohort included 17 (12.8%) of previous > 50 Gy2 EQD2 cases, but severe toxicities were not observed. According to two retrospective studies, poor dosimetric coverage of the GTV (D_min_, D_98_, and D_95_) may be important risk factors for local failure [44, 45]. However, the trade-off between sacrificing coverage of the target volume to meet critical OAR constraints, such as the spinal cord and esophagus, is more important for patients with a high-dose irradiation history.

SBRT is also an expected strategy to improve disease control and survival outcomes in patients with oligometastatic disease [4]. Although oligo-metastasis and oligo-recurrence have been proposed for many years [28–30, 46], randomized-controlled trials using SBRT have only recently emerged and have achieved positive results. In an open-label randomized phase II SABR-COMET trial, standard palliative radiotherapy was compared to SABR (i.e., SBRT) in 99 patients with 1–5 metastatic lesions and a controlled primary tumor. Five-year OS was significantly greater in the SABR arm than in the palliative radiotherapy arm (42.3% vs. 17.7%; *P* = 0.006) [11]. However, it should be noted that there were three deaths (4.5%) in the SABR arm related to stereotactic treatment [4], and further studies are required to prevent the severe toxicities of performing SBRT for high-risk patients such as those in this study.

We experienced one TRD case in the present study due to esophageal perforation, even though all specified SBRT dose constraints were met. Retrospective assessment of the SBRT dose and cumulative dose initial EBRT and SBRT revealed that the TRD patient received the highest doses compared to other patients. Treatment-related death due to radiation-induced esophageal toxicity after re-irradiation SBRT is a rare complication, and we found only six papers (seven patients) as far as we reviewed pertinent literature (Table S5): three cases were treated for lung tumors, and four were spine SBRT. TRD occurred in one re-irradiation case and the rest in the initial RT cases [21, 24, 47–51]. The associated risk factors for severe esophageal toxicity identified were esophageal dose, chemotherapy use, re-irradiation, and iatrogenic esophageal manipulation (e.g., biopsy). The patients in our study received higher cumulative esophageal doses, both D_max_ and D_1cc_, compared to those reported for evaluable TRD patients. Concurrently, 71.4% of the patients had chemotherapy for active systemic disease. Although such factors were associated with an increased risk of severe esophageal toxicities, severe ones were absent in our cohort except for one case that irradiated the highest dose. A possible explanation for the acceptable incidence of severe esophageal toxicity from re-irradiation may be the long interval between the initial EBRT and re-irradiation (median 13.7 months). The risk of toxicity increases with shorter intervals between re-irradiations, which is supported both at the experimental level and in several re-irradiation studies [52–54]. The cumulative dose of our TRD patient received the highest cumulative doses (D_max_ of 227 Gy_3_ BED, D_0.035 cc_ of 209 Gy_3_ BED, and D_1cc_ of 175 Gy_3_ BED, Table 3), so careful consideration is critical in case of re-irradiation for patients with high-dose EBRT history. According to the results of the esophageal dose assessment, the following esophageal cumulative dose constraints are recommended in addition to dose constraints of only the SBRT to reduce the risk of severe esophageal toxicity: D_max_ < 203 Gy_3_ BED, D_0.035 cc_ < 187 Gy_3_ BED, D_1cc_ < 167 Gy_3_ BED, which were based on the highest esophageal cumulative doses in the patients other than TRD. These cumulative esophageal dose constraints, appropriate time to re-irradiation should be determined by each patient and will be updated as more toxicity data are accumulated in the future. The present data did not include severe toxicities of other OARs (pharynx, trachea, carotid arteries, and aorta), and further data are needed to know whether cumulative dose limits of these OARs exist at higher levels.

There are several possible significant limitations in this study. The data were a small sample size from a single institution. Our data were obtained by retrospective observation, and the timing of the evaluation varied. Our recommended cumulative esophageal dose should be validated in future prospective studies with a larger sample size. Also, we note that the study population included 15 patients (71%) who received definitive initial EBRT, not palliative treatment.

In conclusion, in cervical thoracic MESCC patients with a history of irradiation of 50 GyEQD2 or more, SBRT re-irradiation may be expected to have favorable local control similar to that of patients with a lower irradiation history. Cumulative dose constraints for the esophagus (D_max_ < 203 Gy_3_ BED, D_0.035 cc_ < 187 Gy_3_ BED, D_1cc_ < 167 Gy_3_ BED) were key findings for safer treatment.

### Supplementary Information

Below is the link to the electronic supplementary material.

Supplementary file1 (DOCX 32 KB)
