# Supplementary material for: Re-irradiation spine stereotactic body radiotherapy following high-dose conventional radiotherapy for metastatic epidural spinal cord compression: a retrospective study
Source: Jpn J Radiol. 2024 Feb 28;42(6):662–72. doi: 10.1007/s11604-024-01539-x (PMC11139739; doi:10.1007/s11604-024-01539-x)
Supplement: Supplementary file 1 — Supplementary file1 (DOCX 32 KB) [file 11604_2024_1539_MOESM1_ESM.docx]

**Supplementary files**

**Table S1**. **Dose specification for the planning target volume**

| Target | Coverage |
| --- | --- |
| PTV_eval_ D_95_ | >70% of the prescribed 24 Gy  (As high as possible under satisfying the constraints for the surrounding organs at risk) |
| PTV_eval_ D_50_ | >110% and <115% of the prescribed 24 Gy |
| PTV D_0.035cc_ | <140% of the prescribed dose |

PTV: planning target volume, PTV_eval_: the volume obtained by subtracting the spinal cord planning organ at risk volume from the PTV, D_95_: the percentage of the minimum dose that covered 95% of the target volume, D_50_: the percentage of the minimum dose that covered 50% of the target volume, D_0.035cc_: the maximum dose that covered 0.035cc of the target volume

**Table S2**. **Dose constraints for specified organs at risk**

| Organs | Initial EBRT dose, Gy_2_ BED2 | Constraints (per 2 fx) |
| --- | --- | --- |
| Spinal cord PRV | ≤90  90–91  91–92  92–93  93–94  94–95  95–96  96–97  97–98  98–99  99–100  >100 Gy | D_0.035cc_<12.2 Gy  D_0.035cc_<12.1 Gy  D_0.035cc_<12.0 Gy  D_0.035cc_<11.8 Gy  D_0.035cc_<11.7 Gy  D_0.035cc_<11.5 Gy  D_0.035cc_<11.4 Gy  D_0.035cc_<11.2 Gy  D_0.035cc_<11.1 Gy  D_0.035cc_<10.9 Gy  D_0.035cc_<10.8 Gy  D_0.035cc_<10.8 Gy |
| Pharynx | Constant regardless of irradiation history | D_1cc_<20 Gy |
| Larynx | Constant regardless of irradiation history | D_1cc_<20 Gy |
| Esophagus | Constant regardless of irradiation history | D_1cc_<20 Gy |
| Trachea | Constant regardless of irradiation history | D_1cc_<20 Gy |
| Main bronchus | Constant regardless of irradiation history | D_1cc_<20 Gy |
| Stomach | Constant regardless of irradiation history | D_1cc_<20 Gy |
| Lungs | Not defined | Not defined |
| Carotid arteries | Constant regardless of irradiation history | D_1cc_<20 Gy |
| Aorta | Constant regardless of irradiation history | D_1cc_<20 Gy |

PRV: planning target volume, D_0.035cc_: the maximum dose that covered 0.035cc of the target volume, D_1cc_: the maximum dose that covered 1cc of the target volume fx: fractions, EBRT: external beam radiation therapy, BED2: biological effective dose with the α/β ratio of 2

* Unspecified normal tissue was suggested D_1cc_<20 Gy like other OARs but spinal cord

**Table S3**. **Cumulative dose assessment of organs at risk other than the esophagus**

|  | Cumulative dose, maximum value [IQR] | | |
| --- | --- | --- | --- |
|  | D_max_ | D_0.035cc_ | D_1cc_ |
| Spinal cord (N = 11) |  |  |  |
| EQD2  α/β = 2, Gy_2_  BED  α/β = 2, Gy_2_ | 65.5 [56, 62]  131 [112, 124] | 61.0 [52, 56]  122 [104, 112] | NA  NA |
| Pharynx, Larynx (N = 6) |  |  |  |
| EQD2  α/β = 2, Gy_2_  α/β = 3, Gy_3_  α/β = 10, Gy_10_  BED  α/β = 2, Gy_2_  α/β = 3, Gy_3_  α/β = 10, Gy_10_ | 129 [82, 111]  120 [76, 104]  102 [67, 89]  258 [164, 222]  200 [127, 173]  122 [80, 106] | 124 [76, 106]  117 [70, 101]  100 [66, 87]  248 [152, 212]  194 [117, 168]  120 [79, 104] | 113 [63, 92]  107 [63, 89]  95 [63, 80]  226 [126, 184]  178 [104, 148]  114 [75, 96] |
| Bronchus, Trachea (N = 14) |  |  |  |
| EQD2  α/β = 2, Gy_2_  α/β = 3, Gy_3_  α/β = 10, Gy_10_  BED  α/β = 2, Gy_2_  α/β = 3, Gy_3_  α/β = 10, Gy_10_ | 127 [72, 109]  118 [67, 94]  97 [70, 86]  254 [144, 218]  197 [111, 157]  161 [84, 103] | 114 [70, 102]  114 [66, 89]  95 [67, 84]  228 [140, 204]  190 [110, 148]  114 [80, 101] | 101 [65, 92]  100 [50, 79]  87 [54, 76]  202 [130, 184]  167 [83, 131]  104 [65, 91] |
| Carotid arteries (N = 11) |  |  |  |
| EQD2  α/β = 2, Gy_2_  α/β = 3, Gy_3_  α/β = 10, Gy_10_  BED  α/β = 2, Gy_2_  α/β = 3, Gy_3_  α/β = 10, Gy_10_ | 126 [103, 115]  118 [96, 107]  101 [73, 90]  252 [206, 230]  197 [160, 178]  121 [87, 108] | 120 [96, 110]  113 [88, 103]  98 [69, 87]  240 [192, 220]  188 [147, 171]  118 [83, 104] | 104 [71, 93]  100 [67, 89]  90 [58, 81]  208 [142, 186]  167 [111, 148]  108 [70, 97] |
| Aorta (N = 16) |  |  |  |
| EQD2  α/β = 2, Gy_2_  α/β = 3, Gy_3_  α/β = 10, Gy_10_  BED  α/β = 2, Gy_2_  α/β = 3, Gy_3_  α/β = 10, Gy_10_ | 249 [123, 182]  224 [119, 163]  164 [89, 120]  498 [246, 364]  373 [198, 271]  197 [106, 144] | 241 [113, 176]  217 [112, 158]  160 [85, 117]  482 [226, 352]  361 [200, 263]  192 [102, 140] | 197 [90, 159]  180 [88, 144]  141 [73, 107]  394 [180, 318]  300 [147, 240]  169 [87, 128] |

EQD2: equivalent dose at 2 Gy, BED: biological effective dose, IQR: interquartile range, SBRT: stereotactic body radiation therapy, Cumulative dose: total dose accumulated SBRT dose and the initial external beam radiation therapy doses, D_max_: the maximum dose at one point of the target volume, D_0.035cc_: the maximum dose that covered 0.035cc of the target volume, D_1cc_: the maximum dose that covered 1cc of the target volume

**Table S4. Studies in re-irradiation Spine SBRT following conventional radiotherapy**

| Authors, Year | Study type | Patients  (targets) | Median initial RT dose (range) | Re-irradiation dose | 1-year LC | VCF | G4–5 AEs |
| --- | --- | --- | --- | --- | --- | --- | --- |
| Sahgal, 2009 [31] | Retrospective | 25 (37) | 36 Gy/14 fx | 24 Gy/3 fx | 96% | NA | 0 |
| Choi, 2010 [32] | Retrospective | 42 (51) | 40 Gy | 20 Gy/2 fx | 73% | NA | Myelopathy  (G4: 1) |
| Garg, 2011 [33] | Prospective | 59 (63) | 33 Gy | 27 Gy/3 fx  30 Gy/5fx | 76% | NA | 0 |
| Damast, 2011 [34] | Retrospective | 92 (92) | 30 Gy | 20–30 Gy/5 fx | 66% | 9.8% | 0 |
| Mahadevan, 2011 [35] | Retrospective | 60 (81) | 30 Gy | 25–30 Gy/5 fx | NA | NA | 0 |
| Ahmed, 2012 [36] | Prospective | (22) | 30 Gy/ 10 fx | 24 Gy/3 fx | 83% | 4.5% | 0 |
| Chang, 2012 [37] | Retrospective | 54 (54) | 39 Gy | 20.6 Gy/1 fx | 81% | <10% | 0 |
| Thibault, 2014 [38] | Retrospective | 11(11) | 30 Gy | 24 Gy/2 fx | 83% | 16% | 0 |
| Hashmi, 2016 [39] | Retrospective | 215 (247) | 30 Gy/10 fx | 18 Gy/1 fx | 83% | 4.5% | 0 |
| Boyce-Fappiano, 2017 [40] | Retrospective | 162 (237) | 30 Gy/10 fx | 16 Gy/1 fx | NA | 9.3% | 0 |
| Ito, 2018 [41] | Retrospective | 28 (28) | 30 Gy/10 fx | 24 Gy/2 fx | 70% | 10.7% | 0 |
| Sasamura [42] | Retrospective | 40 (42) | 30 Gy/10 fx | 25 Gy/5 fx | 67% | 2.4% | 0 |
| Ito, 2021 [43] | Retrospective | 123 (133) | 30–40 Gy_2_ EQD2  >50 Gy_2_: 13% | 24 Gy/2 fx | 74% | 13.8% | 0 |
| Current study | Retrospective | 21 (21) | 60 Gy_2_ EQD2  (50–105 Gy_2_) | 24 Gy/2 fx | 71% | 23.8% | Esophagitis  (G5: 1) |

RT: radiotherapy, LC: local control, VCF: vertebral compression fracture, AEs: adverse events, fx: fractions, EQD2: equivalent dose at 2 Gy

**Table S5. Studies including radiation-induced grade 4–5 esophageal toxicity after re-irradiation SBRT**

| Authors, Year | Patients (targets) | Target | RT dose | G4 | G5 | D_max_ Gy_3_ BED | D_1cc_ Gy_3_ BED | Risk Factor |
| --- | --- | --- | --- | --- | --- | --- | --- | --- |
| Onimaru, 2003 [48] | Unirradiated: 32 (39) | Lung | 48 Gy/8 fx | 0 | 1 | 157 (G5) | 118 (G5) | Esophageal dose |
| Le QT, 2006 [49] | Unirradiated: 26 (26)  Irradiated: 6 (6) | Lung | Up to 25 Gy/1 fx | 0 | 1 | NA | NA | Esophageal dose  Re-irradiation  Chemotherapy use |
| Gomez, 2009 [50] | Unirradiated: 114 (119) | Spine | Up to 24 Gy/ 1fx | 1 | 0 | NA | 221 (G4) | Esophageal dose |
| Abelson, 2012 [51] | Unirradiated: 31 (31) | Lung/Spine | 25 Gy/1 fx  24 Gy/2 fx | 0 | 2 | 168 (G5) | 139 (G5) | Chemotherapy use |
| Cox, 2012 [24] | Unirradiated: 182 (204) | Spine | 24 Gy/1 fx | 4 | 1 | NA | NA | Chemotherapy use  Iatrogenic manipulation |
| Cassidy, 2022 [21] | Unirradiated: 1 (1) | Spine | 35 Gy/5 fx | 0 | 1 | 138 (G5) | 122 (G5) | Esophageal dose |
| Current study | Irradiated: 21 (21) | Spine | 24 Gy/2 fx | 0 | 1 | 127 (G5)  227 * | 83 (G5)  175 * | Esophageal dose  Cumulative esophageal dose  Chemotherapy use |

RT: radiotherapy, fx: fractions, BED: biological effective dose

* Cumulative esophageal dose
